# The relation between plasma tyrosine concentration and fatigue in primary biliary cirrhosis and primary sclerosing cholangitis

**DOI:** 10.1186/1471-230X-5-11

**Published:** 2005-03-24

**Authors:** Pieter CJ ter Borg, Durk Fekkes, Jan Maarten Vrolijk, Henk R van Buuren

**Affiliations:** 1 Department of Gastroenterology and Hepatology, Erasmus MC, Rotterdam, The Netherlands; 2 Departments of Psychiatry and Neuroscience, Erasmus MC, Rotterdam, The Netherlands

## Abstract

**Background:**

In primary biliary cirrhosis (PBC) and primary sclerosing cholangitis (PSC) fatigue is a major clinical problem. Abnormal amino acid (AA) patterns have been implicated in the development of fatigue in several non-hepatological conditions but for PBC and PSC no data are available. This study aimed to identify abnormalities in AA patterns and to define their relation with fatigue.

**Methods:**

Plasma concentrations of tyrosine, tryptophan, phenylalanine, valine, leucine and isoleucine were determined in plasma of patients with PBC (n = 45), PSC (n = 27), chronic hepatitis C (n = 22) and healthy controls (n = 73). Fatigue and quality of life were quantified using the Fisk fatigue severity scale, a visual analogue scale and the SF-36.

**Results:**

Valine, isoleucine, leucine were significantly decreased in PBC and PSC. Tyrosine and phenylalanine were increased (p < 0.0002) and tryptophan decreased (p < 0.0001) in PBC. In PBC, but not in PSC, a significant inverse relation between tyrosine concentrations and fatigue and quality of life was found. Patients without fatigue and with good quality of life had increased tyrosine concentrations compared to fatigued patients. Multivariate analysis indicated that this relation was independent from disease activity or severity or presence of cirrhosis.

**Conclusion:**

In patients with PBC and PSC, marked abnormalities in plasma AA patterns occur. Normal tyrosine concentrations, compared to increased concentrations, may be associated with fatigue and diminished quality of life.

## Background

Primary biliary cirrhosis (PBC) and primary sclerosing cholangitis (PSC) are chronic cholestatic liver diseases characterized by a usually slowly progressive course [1,2]. Many patients remain in good clinical condition for many years but may suffer from fatigue interfering with normal activities and general quality of life during a significant part of their life [3-5]. Fatigue is not related to the severity or activity of the liver disease, and its pathophysiology remains unknown [3,4,6]. In several non-hepatological conditions amino acids, in particular tryptophan and tyrosine, have been reported to be involved in the pathophysiology of fatigue [7,8]. Plasma amino acid abnormalities have been studied extensively in patients with liver failure and hepatic encephalopathy [9]. In patients with less advanced liver disease of various etiologies, significant differences with respect to plasma amino acid concentrations and tyrosine metabolism have been reported in comparison with control individuals. These studies were performed more than two decades ago, at a time when fatigue had not been identified as a significant problem in cholestatic liver disease. Thus far, the potential role of abnormalities in amino acid metabolism in fatigue associated with cholestatic liver disease has not been evaluated and relevant data in PSC are completely lacking.

The present study aimed to identify abnormalities in plasma concentrations of several amino acids and their relation to fatigue and quality of life in patients with PBC and PSC.

## Methods

The study was approved by our institution's medical ethics committee and informed consent was obtained from each patient. Patients with a diagnosis of PBC (45) or PSC (27) visiting the hepatology outpatient clinic of the Erasmus Medical Center between October 2001 and June 2002 were invited to participate. Exclusion criteria were an age of less than 18 years and incomplete understanding of the Dutch language. As controls with respect to amino acid concentrations, a group of 22 patients with untreated chronic hepatitis C virus infection (HCV) and a group of 73 healthy individuals were included. Fatigue in patients with PBC and PSC was quantified using a visual analogue scale (VAS) and the Fisk fatigue severity scale (FFSS). The FFSS has been validated for use in PBC, and quantifies fatigue in a physical, social and cognitive domain [6,10]. Quality of life was quantified using the SF-36, a widely used quality of life questionnaire [11]. These questionnaires were also obtained from a separate group of 18 age and sex-matched controls, because no questionnaires could be obtained from the previous group of 73 healthy individuals in whom amino acid concentrations were determined. Total serum bilirubin, serum albumin, prothrombin time and serum activities of alkaline phosphatase (AP) and aspartate aminotransferase (AST) were obtained as markers of disease activity and severity. The presence of cirrhosis was determined on the basis of histological and, if not available, clinical criteria (ultrasound findings compatible with cirrhosis if supported by the presence of thrombocytopenia or esophageal varices).

### Amino acid measurement

Immediately after the venapuncture plasma was prepared by a 20 min centrifugation step at 2650 g and stored at -80°C. The amino acids phenylalanine, tyrosine, tryptophan, isoleucine, leucine and valine were measured by means of high-performance liquid chromatography as described elsewhere [12]. The tryptophan ratio, which is the ratio of tryptophan to the summed concentrations of phenylalanine, tyrosine, isoleucine, leucine and valine, was determined as a measure for central availability of tryptophan for serotonin synthesis. The tyrosine ratio was determined as a measure for central availability of tyrosine for dopamine and norepinephrine synthesis and was calculated as the concentration of tyrosine divided by the sum of the concentrations of phenylalanine, tryptophan, isoleucine, leucine and valine.

### Statistics

Testing for differences between groups was performed using Student's t-test and the χ^2 ^test. Correlations were tested using Pearson's correlation method. The normality of amino acid distributions was assessed visually using histograms, and non-parametric tests were used where appropriate. The relations between amino acid concentrations and fatigue scores were tested by calculating correlation coefficients for VAS and FFSS domain scores and plasma amino acid concentrations. In these tests, a p-value <0.01 was considered to be statistically significant. In order to quantify the impact of the differences in amino acid on fatigue, for those amino acids which significantly correlated with fatigue, patients were divided into groups with amino acid concentrations within the 95% confidence interval for healthy controls and patients with concentrations outside this range. Testing for differences in fatigue, quality of life and laboratory parameters between these two groups was performed using Student's t-test. Multivariate regression analysis including the biochemical tests of disease activity and severity and the presence of histological or clinical cirrhosis was performed in order to assess the independent association of amino acid abnormalities and fatigue. In all tests other than the correlation tests, a two-sided p-value <0.05 was considered statistically significant. Statistical analyses were performed using SPSS (Version 9.0, SPSS Inc, Chicago, IL, U.S.A).

## Results

### Patient characteristics

Patient characteristics for patients with PBC and PSC are shown in table 1. As was expected because of the unbalanced sex distribution in these diseases, the majority of patients with PBC were female and the majority of patients with PSC were male. The frequency of cirrhosis, serum bilirubin and albumin and serum activities of alkaline phosphatase, AST and ALT did not significantly differ for patients with PSC or PBC.

### Amino acids in patients and controls

Table 2 shows the plasma concentrations of amino acids and the tryptophan and tyrosine ratio's for patients with PBC, PSC, HCV and healthy controls. Plasma concentrations of the aromatic amino acids tyrosine and phenylalanine were increased in patients with PBC, whereas in HCV only tyrosine concentration was increased compared to controls.

In PSC, neither of the aromatic amino acids was increased. Tryptophan concentration was decreased in patients with PBC and HCV. Plasma concentrations of the branched chain amino acids valine, isoleucine and leucine were significantly lower in both patients with PBC and PSC. The tryptophan ratio was significantly decreased in patients with PBC and HCV. The tyrosine ratio was significantly increased in all three patient groups.

Within the group of healthy controls, no differences in amino acid concentrations were found for different age groups or sex.

### Amino acids and markers of disease activity and severity

In patients with PBC, significant inverse correlations were present between the branched chain amino acids valine (p = 0.002), isoleucine (p = 0.006) and leucine (p = 0.007) and total serum bilirubin concentrations. Plasma concentrations of the aromatic amino acids tyrosine (p < 0.001) and phenylalanine (p = 0.003) correlated inversely with serum albumin concentrations. There was a significant inverse correlation between plasma valine and the serum activity of AST (p = 0.005). Patients with cirrhosis had significantly increased tyrosine (p = 0.004) and phenylalanine (p = 0.03) concentrations and an increased tyrosine ratio (0.004) compared to non-cirrhotics.

However, all differences in amino acid concentrations retained their significance in when only patients without cirrhosis and with normal bilirubin and albumin were compared to healthy controls.

In patients with PSC, no significant correlations were found between any of the markers of disease activity or severity and fatigue or quality of life.

Patients with PSC and inflammatory bowel disease had significantly decreased concentrations of valine, isoleucine and leucine compared to patients with PSC alone (p = 0.02). The concentrations of tyrosine, phenylalanine and tryptophan were not significantly different.

### Amino acids, fatigue and quality of life

In patients with PBC a significant negative correlation was found between tyrosine concentrations and all fatigue tests. In addition, in these patients a significant negative correlation between tryptophan concentrations and the cognitive domain of the FFSS was found, whereas trends towards significant correlations were found for the other FFSS domains. For the other amino acids, no correlations with fatigue were found (Table 3). In patients with PSC, no significant correlations between amino acids and fatigue were found.

**Figure 1:**
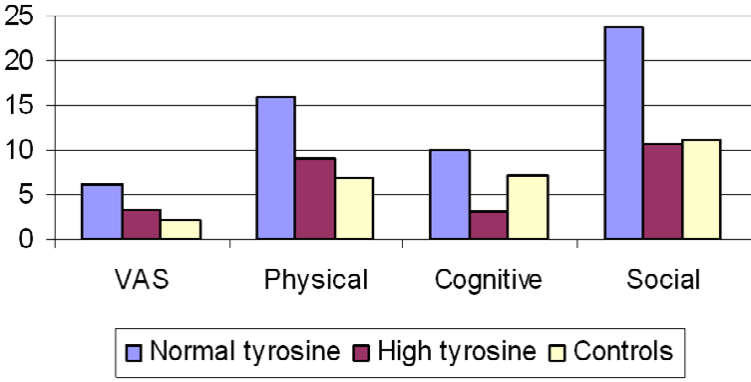
**Fatigue and tyrosine. **Mean VAS scores and scores of the physical, cognitive and social domains of the FFSS for PBC patients with normal tyrosine concentrations (blue), high tyrosine concentrations (red) and controls (yellow).

**Figure 2:**
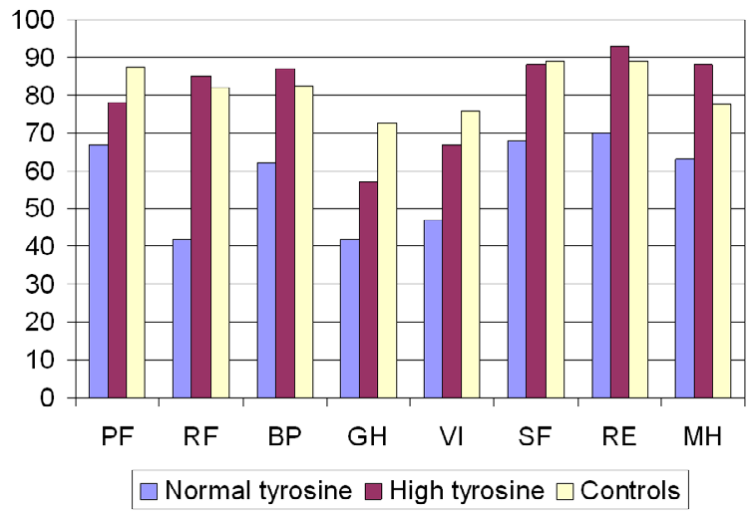
**Quality of life and tyrosine. **Mean SF-36 scores for PBC patients with normal tyrosine concentrations (blue), high tyrosine concentrations (red) and controls (yellow). PF = Physical Functioning, RF = Role Functioning Physical, BP = Bodily Pain, GH = General Health, VI = Vitality, SF = Social Functioning, RE = Role Emotional Functioning, MH = Mental Health.

Comparing PBC patients with normal tyrosine concentrations with patients with increased concentrations resulted in significant differences in VAS (p = 0.03), all domains of the FFSS (p = 0.03, p < 0.001 and p = 0.01 for the physical, cognitive and social domains, respectively) and the role functioning physical (the extent to which physical health interferes with work or other daily activities) (p = 0.001), bodily pain (p = 0.001), general health (p = 0.03), vitality (p = 0.004), social functioning (p = 0.005), role functioning emotional (the extent to which emotional problems interfere with work or other daily activities) (p = 0.008) and mental health (p < 0.001) domains of the SF-36 (Figures 1 and 2). In order to assess confounding by disease severity or activity, we performed multivariate analyses for the measurements of fatigue in PBC including plasma tyrosine concentrations and those laboratory tests which correlated with the amino acid, as well as the presence of cirrhosis, although these laboratory tests and the presence of cirrhosis themselves did not correlate with fatigue or quality of life. These analyses showed that only the plasma tyrosine concentration, and not the laboratory tests or the presence of cirrhosis was significantly and independently associated with fatigue.

Comparing patients with normal tyrosine concentrations with healthy controls resulted in the following significant differences: VAS (p < 0.001), the physical (p < 0.001) and social (p = 0.004) domains of the FFSS and the physical functioning (p < 0.001), role functioning physical (p < 0.001), bodily pain (p = 0.004), general health (p < 0.001), vitality (p < 0.001), social functioning (p = 0.001), role emotional functioning (p = 0.05) and mental health (p = 0.04) domains of the SF-36. There was no significant difference in the cognitive domain of the FFSS. Comparing patients with increased tyrosine concentrations with healthy controls showed no significant differences in any of the tests except for worse scores in the general health (p = 0.03) and better scores in the mental health (p = 0.02) domains of the SF-36 for patients with high tyrosine concentrations.

The mean VAS scores were 6.1 and 3.3 for patients with normal and increased tyrosine concentrations, respectively (p = 0.01). Patients with a VAS score > 5 had a mean tyrosine concentration of 68 μMol/l, whereas patients with a score < 5 had a mean concentration of 86 (p = 0.02).

Tests for differences in fatigue for patients with normal or decreased tryptophan concentrations did not show significant differences between the two groups.

## Discussion

The present study confirms previous findings that significant differences in plasma amino acid concentrations between patients with PBC and healthy controls do exist [13,14]. We found increased concentrations of the aromatic amino acids tyrosine and phenylalanine and decreased concentrations of tryptophan and the branched chain amino acids valine, isoleucine and leucine. Tyrosine concentration correlated with all measurements of fatigue, whereas tryptophan concentrations correlated only with the cognitive FFSS domain. PBC patients with increased tyrosine concentrations reported less fatigue and better quality of life compared to patients with normal concentrations. For PSC, no previous studies on amino acid patterns are available for comparison. We found significant decreases in the plasma concentrations of the branched chain amino acids, and trends towards decreased tryptophan and increased tyrosine and phenylalanine concentrations. However, in contrast to PBC, no relationship with fatigue was found. In addition, we found that valine, isoleucine and leucine concentrations were even lower in patients with PSC and inflammatory bowel diseases than in patients with PSC alone. To our knowledge, no previous data on amino acid concentrations in inflammatory bowel disease are available for comparison.

In several previous studies, mostly on hepatic encephalopathy in patients with advanced cirrhosis, plasma concentrations of amino acids have been studied [9,15]. However, we could identify only two studies including patients with non-cirrhotic PBC. Given the supposedly normal liver function in these patients, these studies somewhat surprisingly found marked differences between patients and controls comparable to those observed in the present study [13,14]. In addition, although the differences appeared to be somewhat smaller, comparable results were obtained in patients with PSC. It remains unclear which mechanisms are responsible for these differences. Although correlations with the markers of disease severity were found, these do not adequately explain the differences in amino acid concentrations, since only a small proportion of the variation in amino acid concentrations could be explained by differences in these markers, and significant differences existed in the majority of patients without cirrhosis and with normal albumin and bilirubin concentrations. Therefore, we suggest other mechanisms, rather than inflammation of the liver or an overall decreased liver function, may be responsible for the noted abnormalities. The nature of these mechanisms, however, remains unknown.

Tyrosine and phenylalanine are mainly metabolized in the liver, suggesting that decreased liver function might result in increased plasma levels. The decreased tryptophan concentrations found in our study might be explained by increased use of tryptophan as a result of immune activation [15,16]. We did not analyze dietary factors that supposedly could influence amino acid concentrations. Previous studies found no evidence to suggest that this is a factor of importance [13,14]. Nearly all patients in the present study were being treated with ursodeoxycholic acid while previous studies reporting comparable plasma amino acid patterns in PBC were performed in the pre-UDCA era [13,14]. Therefore, a role for UDCA in causing these altered patterns seems unlikely.

Fatigue is a significant problem in many patients with PBC and PSC, and has been studied extensively in recent years [3,4,6,17]. However, so far, no specific etiological or pathogenic factors have been identified. Especially, no relation has been found with laboratory parameters for the activity or severity of the disease or histological stage.

An effective medical treatment for fatigue associated with PBC and PSC is not available. Two recent studies specifically addressing PBC-associated fatigue, indicate that treatment with antioxidants is ineffective [18,19].

The present study suggests an association between fatigue and normal tyrosine concentrations in PBC. Concentrations above the 95% confidence interval for healthy controls corresponded with statistically significantly less fatigue and better quality of life scores. Although this suggests that increased tyrosine concentrations may 'protect' against fatigue and normal concentrations may 'cause' fatigue, it may well be that that tyrosine plasma concentration alterations are an epiphenomenon and that both these and fatigue are caused by a so far unknown confounding factor or mechanism. Tyrosine is a precursor in the synthesis of dopa, dopamine, epinephrine and norepinephrine, all of which are important neurotransmitters that might play a role in fatigue. Experimental catecholamine depletion has been reported to worsen fatigue, suggesting that a (relative) lack of tyrosine might be associated with fatigue [20,21]. Further, beneficial effects of tyrosine administration in the prevention of exhaustion and fatigue after physical activity in both animals and humans have been reported [22-24]. Since tyrosine concentrations, and not the tyrosine-ratio was significantly associated with fatigue, a peripheral instead of a central role for tyrosine in the development of fatigue is suggested, which is supported by previous findings supporting peripheral mechanisms in the development of PBC associated fatigue [17]. In addition, other mechanisms, which we did not study, such as abnormalities in the hypothalamo-pituitary-adrenal axis, for example abnormal CRH-release, or manganese homeostasis, might be involved in the development of fatigue in these diseases [25]. In addition, cytokine release as a result of an inflammatory response might also play a role, although studies supporting this hypothesis are lacking. Studies into these mechanisms might therefore be of interest.

It remains unclear why we found a relation between fatigue and tyrosine only in patients with PBC and not in patients with PSC. Although it is likely that fatigue would have a similar etiology in these diseases, the cause of fatigue remains unknown and therefore different mechanisms may occur in these related diseases. Another explanation might be a lack of power to detect a difference in patients with PSC, because less patients with PSC than with PBC were included. On the other hand, although the relation between tyrosine and fatigue was highly significant and occurred for all measures of fatigue, the current finding could be the result of chance or be an epiphenomenon not involved in the pathogenesis of fatigue.

Further studies are therefore required to confirm the present findings and to evaluate the effect of tyrosine suppletion in PBC patients with fatigue.

## Conclusion

In conclusion, we showed that in patients with PBC and PSC, marked abnormalities in plasma amino acids occur. In addition, in patients with PBC, normal tyrosine concentrations, compared to increased tyrosine concentrations, may be associated with fatigue and diminished quality of life. This association was independent from the activity and severity of the disease.

## Competing interests

The author(s) declare that they have no competing interests.

## Authors' contributions

All authors participated in study design and read and approved the final manuscript. PB and HB initiated and coordinated the study, performed patient recruitment and inclusion and prepared the manuscript. JV performed recruitment and inclusion of patients with hepatitis C. PB and HB performed the statistical analysis.

## Pre-publication history

The pre-publication history for this paper can be accessed here:



## Figures and Tables

**Table 1:** Patient characteristics

	PBC (n = 45)	PSC (n = 27)
Age (mean, range in years)	58 (34–78)	45 (23–68)
Sex (male / female)	4/45	19/8
Fatigue (yes / no)	32/15	15/12
Cirrhosis (yes / no)	12 / 33	2 / 25
Inflammatory bowel disease (yes / no)	0 / 45	16 / 11
Total serum bilirubin (median, range in μmol/l)	10 (4–98)	15 (6–37)
Serum albumin (median, range in g/l)	42 (31–48)	42 (33–47)
Prothrombin time (median, range in sec.)	12 (10–18)	13 (11–16)
Serum ALT activity (median, range in U/l)	43 (17–484)	35 (10–662)
Serum AST activity (median, range in U/l)	37 (21–241)	31 (18–338)
Serum Alkaline Phosphatase (median, range in U/l)	154 (68–441)	147 (53–1141)

Normal values: bilirubin <18 μmol/l, albumin 35–50 g/l, ALT < 41 U/l (male) < 31 U/l (female), AST < 37 U/l (male) < 31 U/l (female), alkaline phosphatase < 117 U/l, prothrombin time < 13 sec.

**Table 2:** Amino acid measurements (μMol/l)

		PBC (n = 45)	PSC (n = 27)	HCV (n = 22)	controls
Aromatic amino acids	Tyrosine (mean ± SD)	76 ± 26 p = 0.0002	69 ± 24 p = 0.07	73 ± 25 p = 0.01	62 ± 14.4
	Phenylalanine (mean ± SD)	65 ± 15 p < 0.0001	59 ± 11 p = 0.11	54 ± 8 p = 0.26	56 ± 6.9
	Tryptophan (mean ± SD)	39 ± 8 p < 0.0001	43 ± 11 p = 0.085	40 ± 8 p = 0.0003	46 ± 6.1
Branched chain amino acids	Valine (mean ± SD)	211 ± 47 p = 0.002	208 ± 42 p = 0.002	232 ± 45 p = 0.56	238 ± 41.7
	Isoleucine (mean ± SD)	55 ± 16 p < 0.0001	53 ± 13 p < 0.0001	67 ± 19 p = 0.51	70 ± 18.8
	Leucine (mean ± SD)	107 ± 30 p < 0.0001	101 ± 25 p < 0.0001	119 ± 28 p = 0.053	132 ± 27.1
Calculated ratio's	Tryptophan-ratio (mean ± SD)	7.7 ± 1.5 p = 0.02	8.9 ± 1.8 p = 0.051	7.5 ± 1.0 p = 0.004	8.3 ± 1.14
	Tyrosine-ratio (mean ± SD)	16.3 ± 6.5 p < 0.0001	15.1 ± 5.0 p < 0.0001	14.0 ± 3.5 p < 0.0001	11.4 ± 2.17

**Table 3:** Correlation between amino acid concentrations and fatigue for patients with PBC

		VAS	FFSS, physical domain	FFSS, cognitive domain	FFSS, social domain
Aromatic amino acids	Tyrosine	-0.38 **p = 0.01**	-0.37 **p = 0.01**	-0.40 **p = 0.006**	-0.37 **p = 0.01**
	Phenylalanine	-0.27 p = 0.08	-0.29 p = 0.05	-0.25 p = 0.10	-0.31 p = 0.04
	Tryptophan	-0.25 p = 0.10	-0.36 p = 0.02	-0.42 **p = 0.004**	-0.33 p = 0.03
Branched chain amino acids	Valine	-0.10 p = 0.50	-0.21 p = 0.16	-0.06 p = 0.70	-0.04 p = 0.79
	Isoleucine	-0.04 p = 0.76	-0.09 p = 0.54	0.06 p = 0.70	0.09 p = 0.58
	Leucine	-0.09 p = 0.57	-0.16 p = 0.29	0.01 p = 0.98	0.02 p = 0.89
Calculated ratio's	Tryptophan-ratio	-0.02 p = 0.87	-0.07 p = 0.66	-0.26 p = 0.09	-0.19 p = 0.22
	Tyrosine-ratio	-0.30 p = 0.048	-0.21 p = 0.15	-0.34 p = 0.02	-0.32 p = 0.03
